# Characterization of a Unique Carbohydrate-Binding Module from *Akkermansia muciniphila* that Binds Ganglioside-Associated Glycans

**DOI:** 10.1021/acs.biochem.6c00509

**Published:** 2026-10-15

**Authors:** Janique Miller, Joshua Steward, Davide Rossotto, Omid Haji-Ghassemi, Melanie A. Higgins

**Affiliations:** † Department of Biological Sciences, 8063 The University of Alabama, Tuscaloosa, Alabama 35487,United States; ‡ Department of Biological Sciences, 2129 University of Calgary, Calgary T2N 1N4, Canada

## Abstract

*Akkermansia muciniphila* is a mucin-degrading gut symbiont that promotes host metabolic health through its ability to utilize host-derived glycans. Central to this microbe is its diverse set of glycoside hydrolases (GHs), specifically GHs involved in degrading host glycans like mucin. Here, we characterize an *A. muciniphila* GH from family 123 (AmGH123), that has β-*N*-acetyl-d-galactosaminidase activity, removing terminal β-linked GalNAc from ganglioside-associated glycans similar to other GH123s. In addition, we identify and characterize a unique carbohydrate-binding module from family 32 associated with AmGH123 (AmGH123^CBM32^), representing the first CBM characterized from *A. muciniphila*. AmGH123^CBM32^ selectively binds GalNAc-terminated ganglioside-associated glycans, revealing previously unrecognized ligand specificity for CBM32 members. Structural analysis and site-directed mutagenesis reveal a distinct ligand-binding pocket in AmGH123^CBM32^, highlighting diverse strategies for glycan recognition within this family.

## Graphical abstract

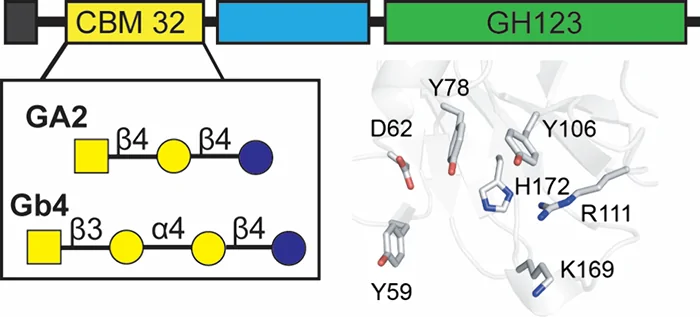

The human gastrointestinal tract harbors a dynamic microbial community that plays a pivotal role in host health. Mucin-degrading bacteria have emerged as a key modulator of gut homeostasis.^1^ Among these, *Akkermansia muciniphila* has garnered attention since its discovery in 2004 due to its ability to colonize the mucus layer and its association with various health benefits.^2,3^ Central to the mucolytic lifestyle of *A. muciniphila* is its repertoire of carbohydrate-active enzymes (CAZymes), particularly glycoside hydrolases (GHs), which catalyze the stepwise degradation of the complex glycan structures decorating mucin glycoproteins. Genomic and biochemical analyses have revealed that *A. muciniphila* encodes many GHs, able to degrade mucin.^4^ However, the full extent of *A. muciniphila* glycan utilization and interaction with other host glycans is largely underexplored.

Glycosphingolipids (GSLs) contribute to the total mass of intestinal glycoconjugates and are involved in host–pathogen interactions, membrane structure, and cell–cell recognition.^5^ Many of them are classified based on their core structures, with GSLs of a common core making up a series. Some series associated with the intestinal tract are denoted as lacto- (Lc), globo- (Gb), and ganglio- (Gg). Altered GSL degradation has been linked to several disorders, and GSLs can also act as receptors for the binding of some enteric toxins, making their microbial degradation biologically important.^6^

*A. muciniphila* contains a GH from family 123 (Amuc_0803; Uniprot B2UQA1; AmGH123). GH123 enzymes are known to remove terminal β-*N*-acetyl-d-galactosamine (GalNAc) with various linkages from GSLs.^7–12^ They are divided into six subfamilies, with AmGH123 belonging to subfamily_1. GH123 has been shown to act on β1,3- and β1,4-linked GalNAc linkages on ganglioside-derived glycans, including GA2 and Gb4.^13^ To further characterize AmGH123, we first produced and purified recombinant full length AmGH123 with the signal peptide removed (AmGH123^FL^) (Figure S1, S2) and tested it against a panel of 4-nitrophenyl (*p*NP) glycosides. As expected, AmGH123^FL^ exhibited robust catalytic activity toward *p*NP-β-d-GalNAc (Figure 1a). We then used this substrate to determine its optimal pH of 7.5 and its *K*_M_ of 248.2 ± 63 μM, *k*_cat_ of 355.45 ± 32 s^–1^, and *k*_cat_ /*K*_M_ of 1.43 ± 0.39 μM^–1^ s^–1^ (Figure 1b,c), which is consistent with other GH123 subfamily enzymes.^8^ Using glycosphingolipid (GSL) associated substrates GM1, gangliotetraose (Galβ-1,3-GalNAcβ-1,4-Galβ-1,4-Glc); GA2, gangliotriose (GalNAcβ-1,4-Galβ-1,4-Glc); Gb4, globo-*N*-tetraose (GalNAcβ-1,3-Galα-1,4-Galβ-1,4-Glc); and Gb3, globotriose (Galα-1,4-Galβ-1,4-Glc), we were also able to confirm its activity on GA2 and Gb4 (Figure 1d,e).

**Figure 1:**
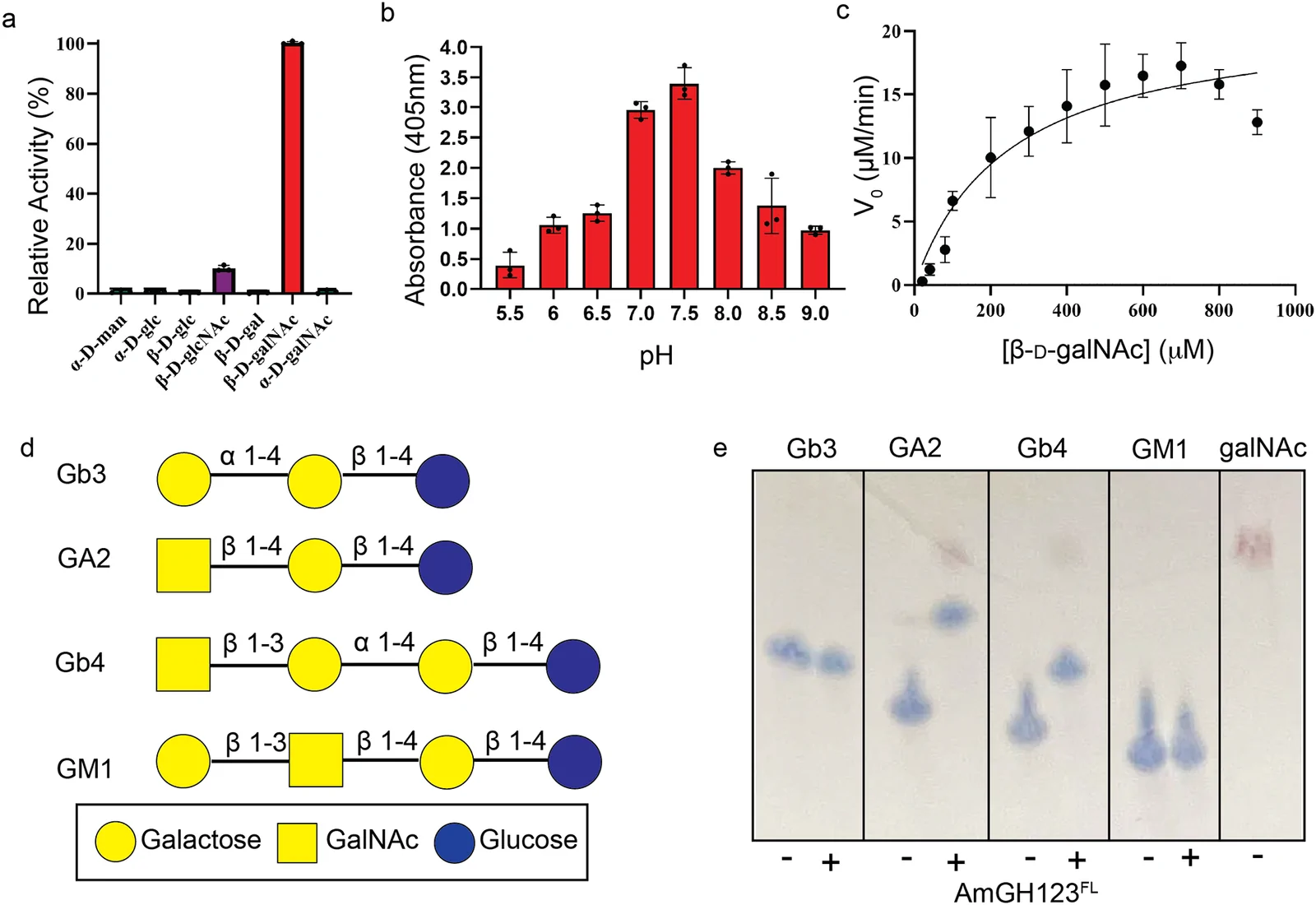
AmGH123^FL^ activity. (a) AmGH123^FL^ activity against different *p*NP-sugars. AmGH123^FL^ (b) pH profile and (c) Michaelis–Menten kinetics with *p*NP-β-d-GalNAc. (d) Structure of GSL-associated glycans. (e) TLC analysis of AmGH123^FL^ activity on GSL associated glycans.

Domain analysis indicates AmGH123 from the N-terminus contains a signal peptide, a carbohydrate-binding module family 32 (AmGH123^CBM32^), a GH123 N-terminal domain and GH123 catalytic module toward the C-terminus (AmGH123^CM^) (Figure S1). Since CBM32 members have not been reported to be associated with other characterized GH123 enzymes, we next sought to determine its ligand specificity. AmGH123^CBM32^ was expressed and purified independently of the catalytic module (residues D43-G180; Figure S2), and tested for binding against gangliosides. Consistent with the catalytic domain’s specificity (Figure 1d), AmGH123^CBM32^ bound GA2 and Gb4 to 0.98 ± 0.5, and 3.6 ± 2.3 mM, respectively, both containing terminal β-GalNAc residues (Table 1; Figure S3). No measurable binding was detected for GM1 or Gb3, indicating that AmGH123^CBM32^ requires terminal GalNAc and cannot bind GSLs terminating in Gal. Weak binding was observed for GalNAc (7.0 ± 2.8 mM), but not GlcNAc. Although CBM32 members exhibit broad ligand specificity, including terminal Glc, GlcNAc, and GalNAc glycans (Table S2),^14^ our results provide, our knowledge, the first evidence of ganglioside-derived glycans as CBM32 ligands and the first report biochemical characterization of a CBM from *A. muciniphila*.

**Table 1:** Binding Affinities of AmGH123^CBM32^ WT, Y59A and K169A Variants Determined by ITC (Figures S3, S6)­^a^

CBM (Cell)	Glycan (Titrant)	[Cell] (mM)	[Titrant] (mM)	Avg *K*_d_* (mM)	Stoichiometry^a^ (*N*)	Avg Δ*G*° (kJ mol ^–1^)	Avg Δ*H*° (kJ mol ^–1^)	Avg *T*Δ*S*° (kJ mol ^–1^)	Repeats
WT	GalNAc	0.2	2.0	7.0 ± 2.8	1^b^	–12 ± 1.0	–90 ± 33	–78 ± 34	3
	GlcNAc	0.2	2.0	ND^c^	ND	ND	ND	ND	2
	GA2	0.03–0.2	0.6–2.0	0.98 ± 0.5	0.93 ± 0.14	–17 ± 1.0	–50 ± 16	–34 ± 17	4
	GM1	0.03–0.2	0.6–2.0	ND^c^	ND^c^	ND^c^	ND^c^	ND^c^	3
	Gb3	0.03–0.2	0.6–2.0	ND^c^	ND^c^	ND^c^	ND^c^	ND^#^	3
	Gb4	0.2	1.0–1.5	3.6 ± 2.3	0.94 ± 0.3^b^	–15 ± 1.7	–54 ± 26	–39 ± 28	3

Y59A	GalNAc	0.2	2.0	ND	ND	ND	ND	ND	2
	GA2	0.2	2.0	ND	ND	ND	ND	ND	2

K169A	GalNAc	0.2	2.0	ND	ND	ND	ND	ND	3
	GA2	0.2	2.0	1.3 ± 0.27	0.92^b^ ± 0.11	–17 ± 0.78	–47 ± 16	–29 ± 17	3

a All measurements were performed at 25 °C (298 K).

b Stoichiometry (*N*) and affinities (*K*_d_) become unreliable for >1 mM affinities because saturation/curvature in the binding isotherm cannot be achieved.

c In some replicates (or all), the stoichiometry (*N*) was forced to be 1 for a better fit.

d ND: Could not be determined due to poor fit/low heats.

Given that ganglioside-derived glycans represent unique ligands for CBM32 members, we next sought to identify the ligand-binding site of AmGH123^CBM32^. We determined the crystal structure of AmGH123^CBM32^ at 2.1 Å resolution. AmGH123^CBM32^ crystallizes with two monomers in the asymmetric unit, each adopting a central β-sandwich surrounded by extended loops of short α-helices (rmsd 0.230 Å over 855 atoms) (Figure 2a; Figure S4). As expected, the closest structural homologues identified by Foldseek^15^ are CBM32 members from *Paenibacillus fukuinensis* (PfGH8^CBM32^; rmsd 1.058 Å over 502 atoms; PDB 4zxe)^16^ and *Persicobacter* sp. CCB-QB2 (PsPL7^CBM32^; rmsd 1.269 Å over 486 atoms; PDB 7d2a).^17^ Notably, PfGH8^CBM32^ is associated with a chitinase (GH8) and binds to the terminal GlcNAc in chitosan, while PsPL7^CBM32^ is associated with a polysaccharide lyase from family 7 (PL7) and binds 4,5-unsaturated mannuronic acid. AmGH123^CBM32^ also contains a Ca^2+^ ion, coordinated by the side chain oxygens of D69 and E175, main chain oxygens of Q71, L66, and V174, and an ordered water (Figure 2b), similar to other CBM32 members.^17–20^

**Figure 2:**
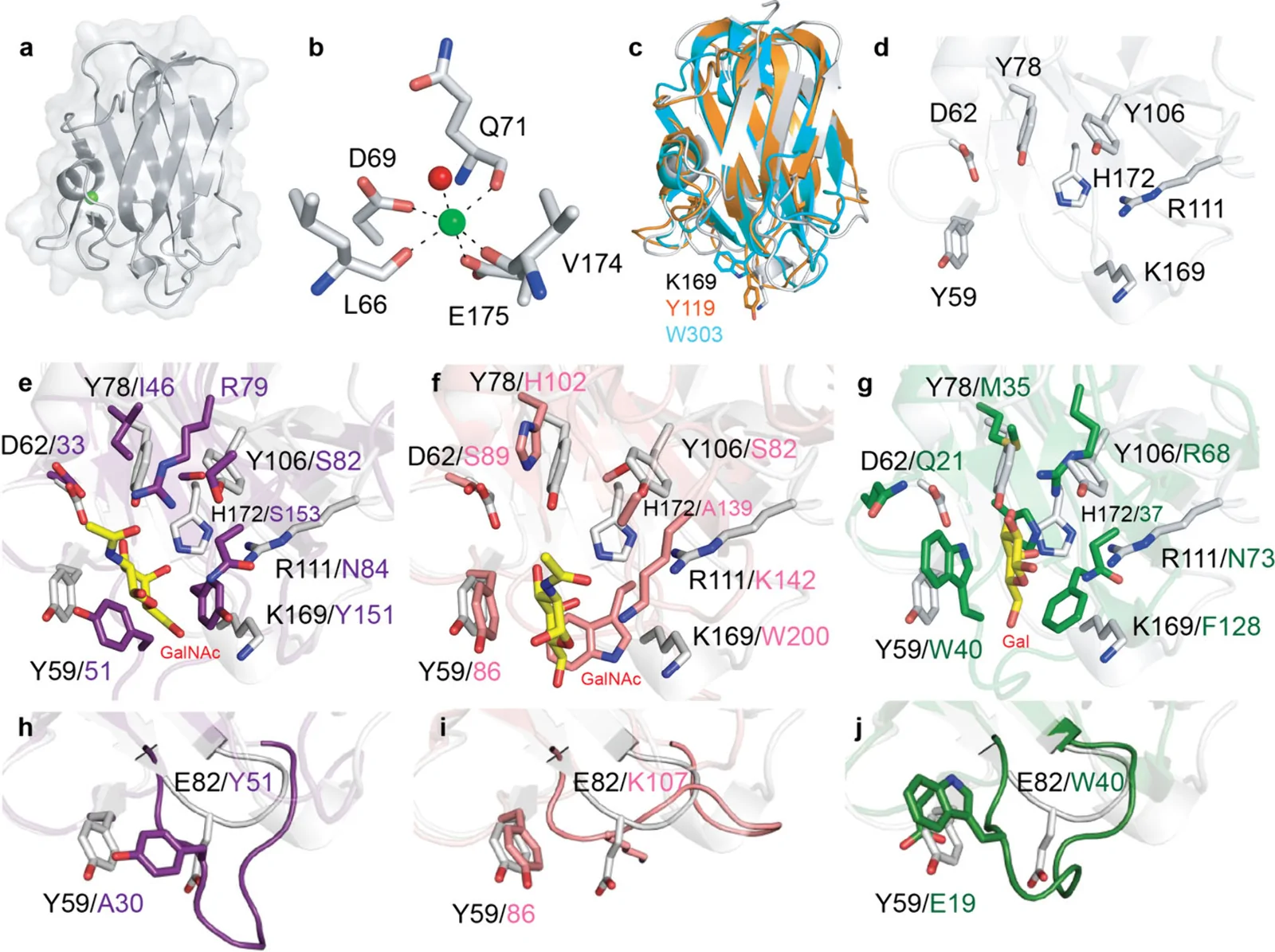
Structure of AmGH123^CBM32^. (a) Overall structure of AmGH123^CBM32^. (b) AmGH123^CBM32^ Ca^2+^ binding site. The Ca^2+^ ion and coordinating water molecule are shown as green and red spheres, respectively. (c) Structural comparison of AmGH123^CBM32^ (gray), PsPL7^CBM32^ (cyan), and PfGH8^CBM32^ (orange), highlighting the residue occupying the conserved aromatic position. (d) Putative ligand-binding site for AmGH123^CBM32^. Comparison of the AmGH123^CBM32^ ligand-binding site (gray) with (e) CpGH31^CBM32–3^ (purple), (f) CpZmpB^CBM32^ (pink), and (g) CpGH33^CBM32^ (green). GalNAc and Gal are represented as yellow sticks. Comparison of the Y59 site from AmGH123^CBM32^ (gray) with (h) CpGH31^CBM32–3^ (purple), (i) CpZmpB^CBM32^ (pink), and (j) CpGH33^CBM32^ (green). Oxygen and nitrogen atoms are colored red and blue, respectively.

We next sought to obtain a structure of AmGH123^CBM32^ in complex with GA2. However, weak binding affinity and fragile crystals prevented us from obtaining a complex structure. Most functional CBM32 members contain a conserved aromatic residue in the ligand binding site, typically located in the extended loops on one end of the central β-sandwich (Table S2). To identify the corresponding site in AmGH123^CBM32^, we overlaid its structure with those of PfGH8^CBM32^ and PsPL7^CBM32^. Surprisingly, the equivalent residue in AmGH123^CBM32^ is not aromatic but corresponds to K169 (Figure 2c). Nevertheless, AmGH123^CBM32^ contains three aromatic residues (Y59, Y78, and Y106) within this region (Figure 2d) and DeepGlycanSite predicted residues in this area to participate in ligand binding (Figure 3a). Together, these observations support this region as the likely ligand-binding site despite the absence of a conserved aromatic residue.

**Figure 3:**
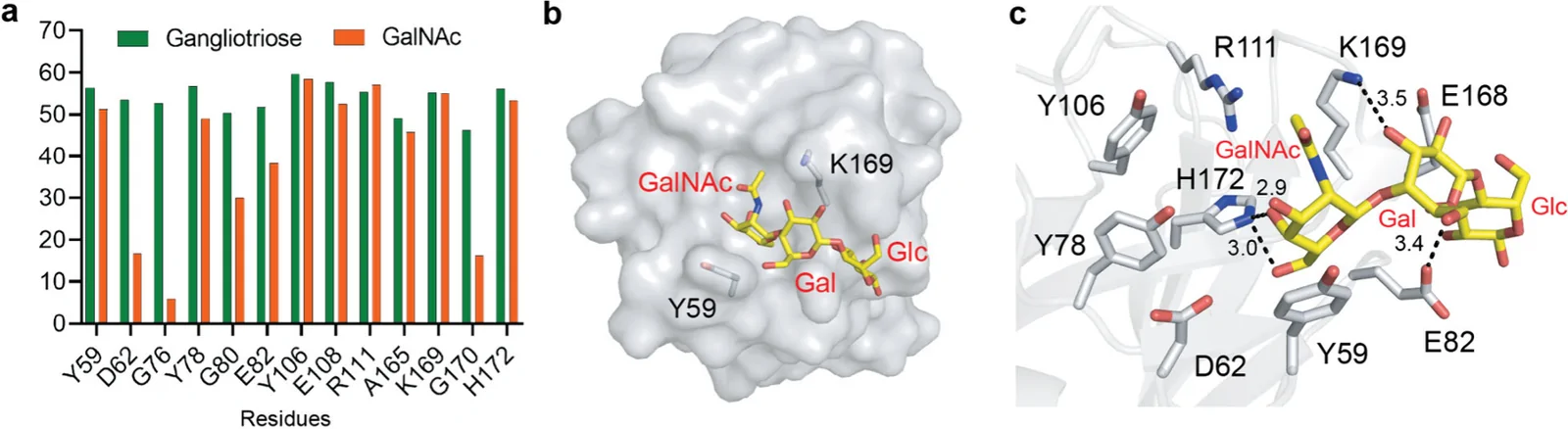
Molecular docking for AmGH123^CBM32^ with GA2. (a) DeepGlycanSite predicted binding residues. (b) Docking of GA2 and AmGH123^CBM32^. GA2 is shown as yellow sticks. (c) Potential ligand-binding interactions for AmGH123^CBM32^ with docked GA2. Potential H-bonding distances are represented in Å. Oxygens and nitrogens are colored red and blue, respectively.

There are four CBM32 members known to bind GalNAc, all from *Clostridium perfringens*. Two are associated with a GH31 that cleaves terminal α-linked GalNAc from mucin-type *O*-glycans (CpGH31^CBM32–2^ and CpGH31^CBM32–3^),^18,21^ one with an *O*-glycopeptidase (CpZmpB^CBM32^),^19^ and one with a sialic acid cleaving GH33 (CpGH33^CBM32^).^20^ Structures containing GalNAc have been solved for CpGH31^CBM32–3^ (PDB 4p5y) and CpZmpB^CBM32^ (PDB 7jnf), while CpGH33^CBM32^ has been solved in complex with Gal (PDB 2v72). We overlaid these ligand binding sites with AmGH123^CBM32^, revealing the presence of the conserved aromatic residue corresponding to K169 for AmGH123^CBM32^ (Figure 2e–g; Table S2). We also observed an aromatic residue occupying a position similar to Y59 from AmGH123^CBM32^. Interestingly, in CpGH31^CBM32–3^ and CpGH33^CBM32^, this aromatic residue originates from a completely different loop than for AmGH123^CBM32^ and CpZmpB^CMB32^. This loop is truncated in AmGH123^CBM32^ and adopts a different orientation for CpZmpB^CBM32^ (Figure 2h–j). Furthermore, this aromatic residue appears to form a π-stacking interaction with the GalNAc or Gal ligand for CpGH31^CBM32–3^, CpZmpB^CBM32^, and CpGH33^CBM32^, suggesting that Y59 may play an important role in ligand recognition by AmGH123^CBM32^. Structural comparisons suggest that AmGH123^CBM32^ may distinguish galacto- from gluco-configured ligands through interactions with the C4 hydroxyl, whereas the molecular basis for its requirement for the GalNAc N-acetyl group remains unclear (Figure S5) and may reflect a distinct binding mode.

We next sought to determine the contribution of Y59 and K169 to AmGH123^CBM32^ ligand binding. Based on structural comparisons with CpGH31^CBM32–3^, CpGH33^CBM32^, and CpZmpB^CBM32^, we hypothesized that Y59 contributes essential π-stacking interaction with the terminal GalNAc ligand. K169 corresponds to the conserved aromatic residue in other CBM32 members and was therefore predicted to contribute to ligand binding. To test these hypotheses, both residues were mutated to alanine, and the variants were produced and purified (Figure S2). Binding analysis revealed that the AmGH123^CBM32‑Y59A^ variant lost heats of binding toward GA2, demonstrating the critical importance of Y59 in ligand recognition (Table 1; Figure S6). By contrast, the AmGH123^CBM32‑K169A^ variant displayed binding affinity toward GA2 comparable to the wild-type (1.3 ± 0.27 mM), suggesting that K169 does not significantly contribute to ligand binding.

We next sought insight into how AmGH123^CBM32^ binds extended glycans. Given the modestly lower *K*_d_ for GA2 than GalNAc alone, we reasoned that the Gal residue in GA2 also contributes to binding. While several CBM32 structures have been solved in complex with β-linked disaccharides, most lack an aromatic residue equivalent to Y59 and primarily interact with only the terminal sugar, suggesting different binding modes (Table S2).

To investigate this further, we performed docking studies using the GA2 trisaccharide (GalNAcβ1→4Galβ1→4Glc) and the AmGH123^CBM32^ crystal structure. Based on structural comparisons, mutagenesis data and DeepGlycanSite residue predictions,^22^ we prioritized orientations in which the terminal GalNAc formed a π-stacking interaction with Y59 (Figure 3a). We therefore used docking as a hypothesis-generating approach, filtering poses for consistency with the experimentally supported Y59 interaction (Figure 3b). In this model, the C4 and C6 hydroxyls of GalNAc potentially form hydrogen bonds with H172, while the Gal residue may form weaker interactions with K169 and E82 (Figure 3c). However, because the K169A variant retained wild-type binding affinity, this predicted interaction is likely weak or nonessential, or alternatively, the docking orientation may not fully represent the native binding mode. Additional studies will be required to determine the ligand-binding mechanism of AmGH123^CBM32^.

Since CBM32 members have not previously been reported in characterized GH123 enzymes (Figure S1),^14^ we assessed the prevalence of CBMs across this GH family using a sequence similarity network (SSN) generated from all GH123 sequences in the CAZy database (Figure 4).^23^ We mapped Pfam domains from known CBM families onto the SSN and identified ten sequences containing CBM32 members, found in three clusters. In addition, two sequences contained a CBM51, two contained a CBM9, and one contained both a CBM6 and a CBM16.

**Figure 4:**
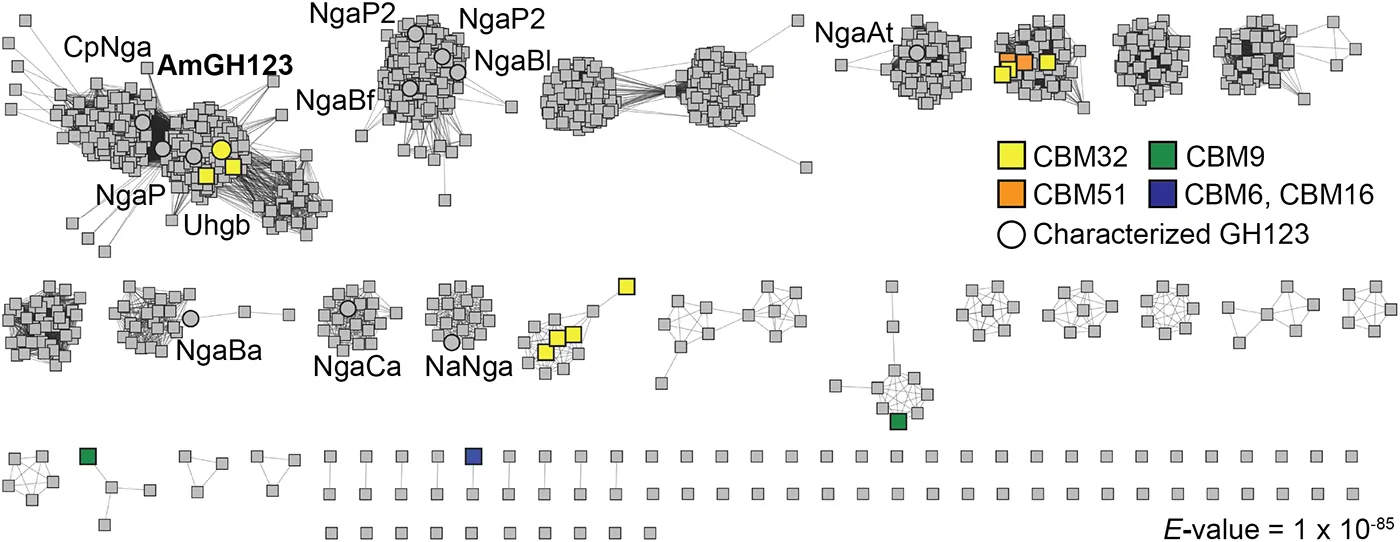
Sequence similarity network for the GH123 sequences.

These findings demonstrate that CBMs are rarely associated with GH123 enzymes and establish AmGH123^CBM32^ as a unique ganglioside-associated glycan-binding module that expands our understanding of glycan recognition and host glycan utilization in *A. muciniphila*. However, as AmGH123 was originally identified through its upregulation in mucin,^24^ we also recognize that AmGH123 could also have mucin degradation activity and further investigation of its degradation capabilities is warranted.

## Supplementary Material



## Notes

Methods and additional figures and tables mentioned in the text (PDF)

## Notes

AmGH123, B2UQA1; PfGH8, Q93IE7; PsPL7, A0ACD6BAS1; CpGH31, A0A0H2YST8; CpZmpB, A0A0H2YN38; CpGH33, Q8XMY5.

## Abbreviations

CBM: carbohydrate-binding module
GH: glycoside hydrolase.
