# Supplementary material for: Characterization of a Unique Carbohydrate-Binding Module from Akkermansia muciniphila that Binds Ganglioside-Associated Glycans
Source: Biochemistry. 2026 Oct 15;65(21):3383–8. doi: 10.1021/acs.biochem.6c00509 (PMC13738339; doi:10.1021/acs.biochem.6c00509)
Supplement: Supplementary file 1 [file bi6c00509_si_001.pdf]

Supporting Information

Characterization of a Unique Carbohydrate-Binding Module from *Akkermansia muciniphila* that binds ganglioside-associated glycans

Janique Miller[1], Joshua Steward[2], Davide Rossotto[1], Omid Haji-Ghassemi[2], Melanie A. Higgins[1]*

[1] Department of Biological Sciences, The University of Alabama, Tuscaloosa, Alabama, USA 35487

[2] Department of Biological Sciences, University of Calgary, Calgary, Alberta, Canada T2N 1N4

* Corresponding author (mahiggins1@ua.edu)

Materials and Methods

GH123 family Sequence analysis

Characterized GH123 sequences were retrieved from the CAZy database (http://www.cazy.org).[1] To verify the boundaries of known domains and predict those of uncharacterized auxiliary domains, the sequences were then analyzed using UniProt[2] and InterPro Scan[3]. A schematic illustration depicting the modular organization of the characterized GH123 enzymes was then generated in Adobe Illustrator 2025 version29.2.1.

Sequence similarity network

A sequence similarity network (SSN) was generated using the Enzyme Function Initiative–Enzyme Similarity Tool (EFI-EST). Protein sequences corresponding to glycoside hydrolase family 123 (GH123) were obtained from the CAZy by downloading the family-specific text file containing all annotated GH123 entries. Accession identifiers were extracted from the downloaded CAZy text file and uploaded to EFI-EST using the Accession ID input option. An alignment score cutoff of 85 was used to generate the SSN. The resulting network file was imported into Cytoscape for visualization and further analysis.

Cloning AmGH123^{FL}, AmGH123^{CBM32} and AmGH123^{CBM32} variants

Akkermansia muciniphila ATCC BAA 835 was obtained from the University of Alabama at Birmingham Microbiota Culture Core and cultured in Brain Heart Infusion supplemented with 0.5% mucin. Colony PCR was used to PCR amplify full length *amgh123^{fl}* (GenBank Accession ACD04636.1) using the primers AmGH123^{FL}-F (5'-GTTAGCCATATGCAGTCCCTGGAGAGGCAT-3') and AmGH123^{FL}-R (5'-GTTAGCCTCGAGGCGGGAAATTTCCGTCGT- 3'), and *amgh123^{cbm32}* using primers AmGH123^{CBM32}-F (5'-GTTAGCCATATGATGCCCCCCCTTCTCATT-3') and AmGH^{CBM32}-R (5'-GTTAGCCTCGAGTTTGCCGGAGCCAAAGTA-3'). The resulting PCR product was purified using the Bio Basic EZ-10 spin column PCR products purification kit then digested for one hour using restriction endonucleases NdeI and XhoI (NEB). The digested product was purified using the Bio Basic EZ-10 spin column PCR products purification kit, then ligated overnight at 16°C using T_4 DNA Ligase (NEB) into the pET22b expression vector. The ligation

was transformed into *E. coli* DH5α then grown on LB agar plates containing 100 µg/mL ampicillin. Plasmids were purified using the Bio Basic EZ-10 spin column Plasmid DNA Miniprep Kit and sequenced by Eurofins Genomics LLC.

To generate the AmGH123^{CBM32} variants, the Quikchange mutagenesis protocol[4] was used with the following primers: AmGH123$^{CBM32-Y59A}$-F (5'-TTCCGGTCATGCGGGCAGTGACC-3') and AmGH123$^{CBM32-Y59A}$ -R (5'-GCGGTTACTTTGGCTC-3') and AmGH123$^{CBM32-K169A}$-F (5'-CGGCAATGAGGCGGGGGGCATC-3') and AmGH123$^{CBM32-K169A}$-R (5'-GCACTATTGCCCAAAAAGTG-3').

Protein production and purification

pET22b:*amgh123^{fl}* and pET22b:*amgh123^{cbm}* were transformed into *E. coli* BL21-Gold (DE3) for protein production. Cultures were grown in autoinduction media containing 20 g/L tryptone, 10 g/L yeast extract, 0.5% (w/v) glycerol, 50 mM NH_4Cl, 2 mM $MgSO_4$, 17 mM KH_2PO_4, 72 mM K_2HPO_4, 0.2% (w/v) lactose, and 0.05% (w/v) glucose at 37 °C supplemented with 100 µg/mL ampicillin and 15 µg/mL tetracycline, for 2 hours at 200 rpm, then shifted to 16 °C for 3 days at 150 rpm. Cells were harvested by centrifugation at 6,000 x g for 10 minutes. Cell pellets were lysed using a chemical lysis method. Briefly, pellets were resuspended in 40 mL of 25% (w/v) sucrose and 50 mM Tris-HCl pH 8.0, then stirred for 10 minutes. Lysozyme (~10 mg) was added and stirred for another 10 minutes. Next, 80 mL of lysis buffer (1% (w/v) deoxycholate, 1% (v/v) Triton X-100, 100 mM NaCl, 20 mM Tris-HCl pH 8.0) was added and stirred for 10 minutes. DNase I (30 µL of 10 mg/mL) and 100 µL of 1 M $MgSO_4$ were then added, followed by a final 10-minute stir. The lysate was then centrifuged at 11,000 × g for 45 minutes at 4 °C.

The soluble portion of the lysate was run over Cytiva Chelating Sepharose™ fast flow resin was charged with 100 mM $NiSO_4$ and equilibrated with binding buffer (20 mM Tris-HCl pH 8.0; 500 mM NaCl). The resin was washed with two column volumes of binding buffer, and the protein was eluted with increasing concentration of imidazole (10 mM-500 mM) in binding buffer. Protein-containing fractions were identified using SDS-PAGE gel electrophoresis and were pooled for further purification using size-exclusion chromatography on a Bio-Rad NG system with a HiLoad™ 26/600 Superdex™ 75pg column (Cytiva Life Sciences™) equilibrated

in 20 mM Tris pH 8.0, 50 mM NaCl. Protein-containing fractions were concentrated using a 10 kDa filtration device from Pall Corporation. Protein concentrations were determined using the calculated extinction coefficients 173,885 $M^{-1}cm^{-1}$ and 37,930 $M^{-1}cm^{-1}$ for AmGH123^{FL} and AmGH123^{CBM32}, respectively.[5]

_p_NP-sugar activity assays

The monosaccharide activity of AmGH123^{FL} was initially tested using the following *p*-nitrophenyl (*p*NP)-linked sugars: *p*NP-α-L-fucopyranoside (*p*NP-α-Fuc), *p*NP-*N*-acetyl-β-D-glucosaminide (*p*NP-β-GlcNAc), *p*NP-β-D-xylopyranoside (*p*NP-β-Xyl), *p*NP-α-D-mannopyranoside (*p*NP-α-Man), *p*NP-β-D-galactopyranoside (*p*NP-β-Gal), *p*NP-*N*-acetyl-α-D-galactosaminide (*p*NP-α-GalNAc) and *p*NP-N-acetyl-β-D-galactosaminide (*p*NP-β-GalNAc) purchased from Biosynth. Each reaction was conducted in a 100 µL volume containing 2 mM *p*NP-sugar, 2 µM AmGH123^{FL}, and 20 mM Tris pH 8.0. Reactions were incubated at 37 °C for 30 minutes, and absorbance was measured at 405 nm using the Molecular Devices SpectraMax M2^{e} to detect the release of *p*-nitrophenol. To determine the optimal pH for enzyme activity, 2 mM *p*NP-β-GalNAc was incubated with 1 µM AmGH123^{FL} with 20 mM buffer mix of MES and Bis-tris propane at pHs 5.5-9.0. The resulting absorbance was measured at 405 nm. Kinetic analysis of AmGH123^{FL} was performed using *p*NP-β-GalNAc at concentrations ranging from 20 µM to 1.4 mM in 20 mM Tris pH 7.5, with 1 nM AmGH123^{FL}. The absorbance at 405 nm was recorded every 30 seconds over a 10-minute period. The slope of the line was recorded from 1.5 minute to 6 minute. The concentration of released *p*-nitrophenol was calculated using an extinction coefficient of 18,300 M^{-1} cm^{-1}.[6] Data were analyzed and visualized using GraphPad Prism 9.

GH123 substrate specificity using thin-layer chromatography

The oligosaccharide activity of AmGH123^{FL} was tested against the following glycosphingolipid associated (GSL) substrates purchased from Biosynth: GM1: gangliotetraose (Galβ-1,3-GalNAcβ-1,4-Galβ-1,4-Glc), GA2: gangliotriose (GalNAcβ-1,4-Galβ-1,4-Glc), Gb4: globo-N-tetraose (GalNAcβ-1,3-Galα-1,4-Galβ-1,4-Glc), and Gb3: globotriose (Galα-1,4-Galβ-1,4-Glc). Each reaction was performed in a 10 µL volume containing 5 mM GSL, 2 µM AmGH123^{FL} enzyme, and 20 mM Tris pH 8.5. Reactions were incubated for 30 minutes at

37 °C. After incubation, 1 μL of each reaction mixture was spotted onto Supelco TLC silica gel 60 F254 plates. The TLC plates were developed using a butanol: acetic acid: water (2:1:1) solvent system and visualized using a diphenylamine-based staining solution containing 4 g diphenylamine, 4 mL aniline, 20 mL 85% phosphoric acid, and 200 mL acetone at 100 °C for 10 minutes.[7]

Isothermal titration calorimetry

AmGH123$^{CBM32\text{-}WT}$, AmGH123$^{CBM32\text{-}Y59A}$, and AmGH123$^{CBM32\text{-}K169A}$ were dialyzed into 250 mM KCl, 20 mM HEPES, 2 mM TCEP, pH 7.4. Glycans were prepared in the final dialysis buffer. All solutions were filtered through 0.22 μm filters and degassed before use. Proteins were loaded into the cell at 30–200 μM, and glycans were titrated from the syringe at 0.6–2.0 mM, as indicated in **Table 1**. Binding experiments used 2 μL injections of glycan into protein, with 90 s spacing and stirring at 125 rpm. Control titrations of glycan into buffer were performed under matched conditions and subtracted from binding runs. Thermograms were integrated and fitted in NanoAnalyze v4.101 using a one-site independent binding model. Because the measured affinities were in the low millimolar range, K_d values are reported as apparent affinities, and fitted N values should not be interpreted quantitatively. Reported values are mean ± SD from 2–4 independent titrations.

AmGH123^{CBM32} crystallization, data processing and structure elucidation

AmGH123^{CBM32} was initially screened at 45 and 60 mg/mL with Microlytic MCSG 1-4 screens using sitting drop vapor diffusion at room temperature, but no crystallization was observed, and most drops remained clear. To facilitate crystallization, the protein buffer was exchanged with deionized water (diH_2O) using a Cytiva PD10 column and screened again at 45 and 60 mg/mL using MCSG screens and PEG/Ion screens (Hampton Research) in the same conditions. A crystal hit was obtained and optimized using hanging drop vapor diffusion at room temperature. The best crystals were grown at 34% (w/v) PEG 3350, 0.2 M sodium phosphate monobasic monohydrate, and 5 mM calcium chloride, using a protein concentration of 45 mg/mL. Crystals were transferred to cryoprotectant (crystallization solution supplemented with 12% ethylene glycol) and flash-frozen in liquid nitrogen. X-ray diffraction data were collected at the Stanford Synchrotron Radiation Lightsource (SSRL), beamline 12-2. Data was processed

with iMosflm[8] and scaled using AIMLESS[9]. Molecular replacement was performed using PHENIX Phaser[10], with an AlphaFold-predicted model as the search model[11]. Initial model building was carried out using phenix.autobuild[12], followed by iterative refinement with phenix.refine[12] and manual model adjustments in Coot[13]. The final structure was validated by MolProbity and Rampage.[14] Data processing and refinement statistics can be found in **Table S1**.

DeepGlycanSite

DeepGlycanSite[15] was used to identify putative binding residues of AmGH123^{CBM32}. The refined AmGH123^{CBM32} crystal structure was processed through DeepGlycanSite's preprocessing pipeline to generate spatial and physicochemical embeddings for each residue. To ensure reproducibility and consistency across computational environments, DeepGlycanSite and all dependencies were containerized using Docker[16], based on the python:3.9-slim-bookworm image.[17] Structured Data Files (SDFs) for two glycan substrates (GA2 and N-acetylgalactosamine (GalNAc) were generated using RDKit (version 2025.03.2) in combination with ChemDraw (version 23.1.2). Binding predictions were conducted separately for each substrate to facilitate comparative analysis of their interaction profiles. DeepGlycanSite generated continuous binding affinity probabilities for each residue, which were visualized and analyzed to identify residues with high predicted binding potential.

A Docker container containing DeepGlycanSite[15] and all necessary dependencies was uploaded to the public Docker Hub registry (https://hub.docker.com/r/drossotto/deepglycansite). Run instructions and data preparation scripts are available on GitHub (https://github.com/drossotto/DGS-Helper). DeepGlycanSite's main application source code is also available on GitHub (https://github.com/xichengeva/DeepGlycanSite).

Docking

SwissDock[18,19] was employed to perform molecular docking of GA2 into the crystal structure of the AmGH123^{CBM32} domain to investigate potential carbohydrate-binding interactions. The 3D structure of GA2 was constructed using ChemDraw (version 23.1.2) and exported in .sdf format for compatibility with docking input requirements. To ensure biologically relevant docking poses, the search space was centered around residues predicted to participate in glycan recognition, as identified by comparative structural analysis and DeepGlycanSite.

Docking calculations were carried out using SwissDock's default parameters under the CHARMM force field, with a focus on generating multiple binding clusters and ranking them by estimated binding free energy. The resulting docking poses were analyzed using the SwissDock web-based viewer to identify high-confidence binding orientations, and the top-ranked conformations were further visualized in PyMOL for structural inspection and figure generation.

Table S1. AmGH123^{CBM32} structure statistics

**Data collection**	
Beamline	SSRL 12-2
Wavelength	0.97946
Space group	P41 21 2
Cell dimensions	
a, *b*, *c* (Å)	81.75, 81.75, 77.08
	90.00, 90.00, 90.00
Resolution (Å)	38.54-2.10 (2.16-2.10)
R_{pim} (within I+/I-)	0.093 (1.222)
I / s*I*	7.9 (2.0)
$CC_{1/2}$	0.992 (0.365)
Completeness (%)	99.7 (96.5)
No. unique reflections	15896 (1234)
Redundancy	17.3 (15.7)
**Refinement**	
Resolution (Å)	2.10
R_{work} / R_{free}	0.1943/0.2343
No. atoms	
Protein	2074
Solvent	150
Ca^{2+}	2
B-factors	
Protein	38.90
Solvent	42.99
Ca^{2+}	40.39
R.m.s. deviations	
Bond lengths (Å)	0.006
Bond angles (°)	0.794
Ramachandran	
Preferred (%)	98.9
Generously allowed (%)	1.1
Disallowed (%)	0.0
TLS groups	17
PDB	36OE

Table S2: CBM32 structure analysis data. Structures highlighted in bold have GalNAc ligands.

PDB	Organism	Assoc. enzyme	Ligand	Rmsd (Å over atoms)[a]	Y59[b]	K169[b]	Ref
3f2z	*Bacteroides fragilis*	None	Unknown	3.294 over 508	E308	T425	N/A
6oe2	*Bacteroides thetaiotaomicron* 7330	None	Unknown	2.324 over 463	E308	T425	[20]
7blg	*Bacteroides thetaiotaomicron* BFG-55	None	Galb3galNAc	3.745 over 557	Q36	F151	N/A
6eon	*Bacteroides thetaiotaomicron* VPI-5482	GH35	Unknown	8.534 over 367	None	S643	[21]
3ggl	*Bacteroides thetaiotaomicron* VPI-5482		Unknown	2.341 over 465	E308	T425	N/A
7wdt	*Bifidobacterium bifidum*	GH20	GlcNAc-6S	1.720 over 423	E62	W183	[22]
9vak	*Bifidobacterium bifidum*	GH20	Galb3glcNac	10.084 over 537	N/A	N/A	[23]
6qep	Bifidobacterium bifidum			14.818 over 465	N/A	N/A	[24]
2ls6	*Clostridium perfringens* ATCC 13124	GH84	Gal	3.129 over 160	D38	T149	[25]
2w1q	*Clostridium perfringens* ATCC 13124	GH84	GlcNAcb3galNac, GlcNAcb2man	2.003 over 386	Q817	W935	[26]
4txw	*Clostridium perfringens* ATCC 13124	GH84		4.555 over 580	V8	R140	N/A
2j1a	*Clostridium perfringens* ATCC 13124	GH84	Galb4glNac	2.455 over 568	W661	F757	[27]
**4lpl**	***Clostridium perfringens* ATCC 13124**	**GH31**	**GalNAc**	**1.774 over 509**	**Y972**	**F1085**	[28]
**4lks**	***Clostridium perfringens* ATCC 13124**	**GH31**	**GalNAc**	**1.705 over 556**	**Y51***	**Y151**	[28]
2vc9	*Clostridium perfringens* ATCC 13124	GH89	Unknown	1.357 over 537	T41	R144	[29]
4a42	*Clostridium perfringens* ATCC 13124	GH89	Not functional	10.960 over 547	N/A	N/A	[30]
4a41	*Clostridium perfringens* ATCC 13124	GH89	Gal	1.947 over 464	Y1395*	F1483	[30]
4a6o	*Clostridium perfringens* ATCC 13124	GH89	GlcNAca4gal	1.596 over 455	Q1223	W1333	[30]
2j1e	*Clostridium perfringens* ATCC 13124	GH89	Galb4glcNAc	2.572 over 578	W661*	F757	[27]
**7jfs**	***Clostridium perfringens* ATCC 13124**	**O-glycopeptidase**	**GalNAc**	**2.490 over 390**	**Y86**	**W200**	[31]

7jfs	*Clostridium perfringens* ATCC 13124	O-glycopeptidase	Not functional	1.810 over 421	Y311	S422	[31]
**2v72**	***Clostridium perfringens* str. 13**	**GH33**	**Gal and GalNAc**	**1.694 over 455**	**W41***	**F128**	[32]
2j7m	*Clostridium perfringens* str. 13	GH84	Fuca2galb4GlcNAc	2.594 over 580	W661	F757	[27]
6tr3	*Mediterraneibacter gnavus* RI1	GH29	Unknown	3.064 over 300	R403	D427	[33]
1eut	*Micromonospora viridifaciens*	GH33	Unknown	2.946 over 548	W542*	Y637	[34]
7nsn	Neobacillus novalis	GH92	Not functional	13.521 over 481	N/A	N/A	[35]
4zz8	*Paenibacillus fukuinensis*	GH8	GlcNAcb4GlcNAc	1.224 over 468	E14	Y120	[36]
4zxe	*Paenibacillus fukuinensis*	GH8	GlcNAcb4GlcNAc	1.058 over 502	E14	Y119	[36]
7d2a	*Persicobacter* sp. CCB-QB2	PL7	4,5-unsaturated mannuronic acid	1.269 over 486	D200	W303	[37]
9g8h	*Pseudarthrobacter siccitolerans*	Galactose oxidase	Unknown	1.736 over 319	Y184*	W277	N/A
2xqx	*Streptococcus pneumoniae*	GH85	Unknown	2.601 over 433	G822	K937	[38]
7w12	*Vibrio splendidus*	PL7	Unknown	1.774 over 515	D24	W129	[39]
2jda	*Yersinia enterocolitica*	None	Repeating a4-galacturonic acids	1.171 over 531	D19	W125	[40]
5k9h	Unidentified	GH29	Unknown	3.343 over 269	None	E425	[41]

[a] RMSD to AmGH123^{CBM32}

[b] corresponds to the AmGH123^{CBM32} residue

* spatially conserved (not from the same loop as Y59)

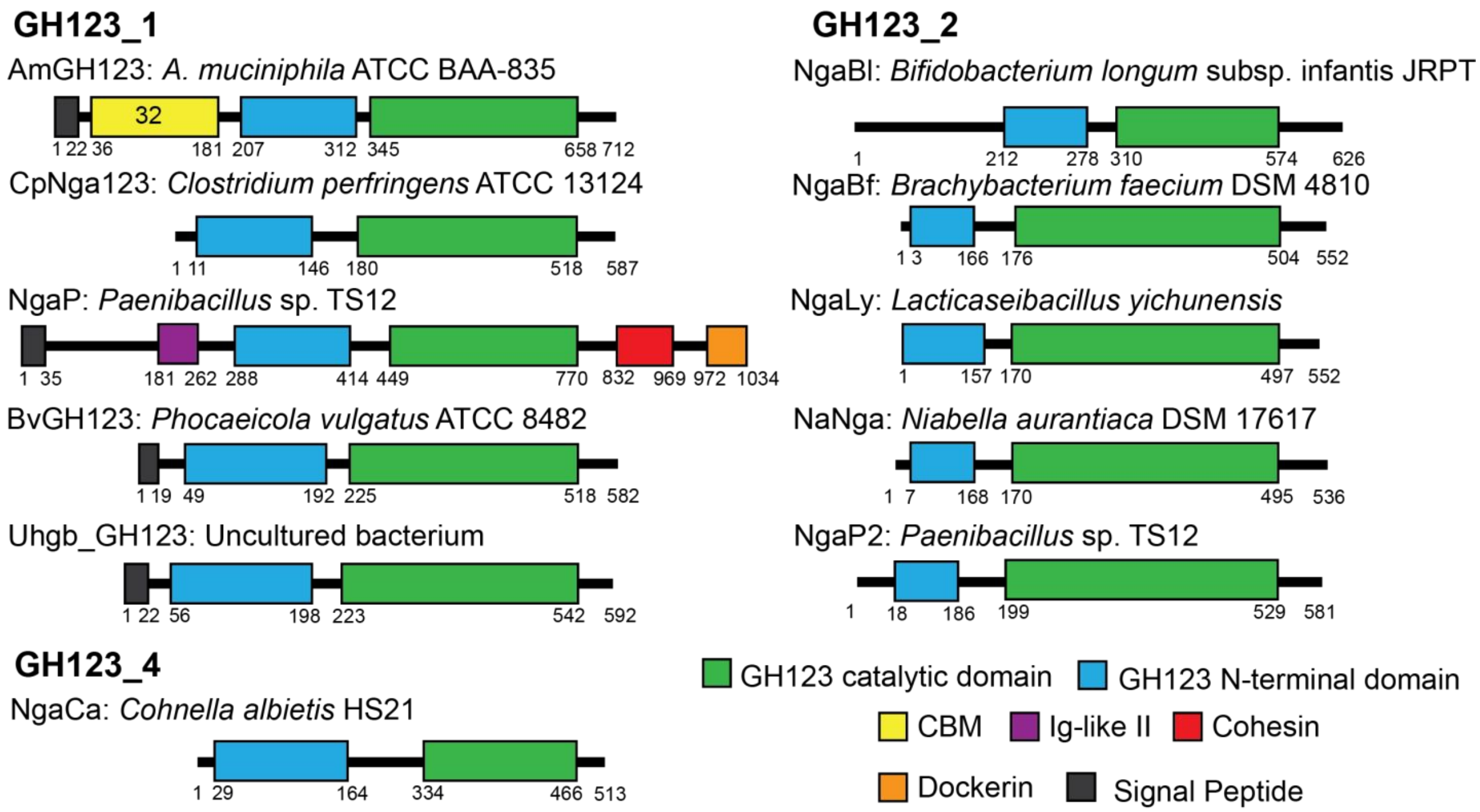

Figure S1: Modular make-up of characterized GH123 enzymes. Domains and boundaries were identified through Interpro Scan[3] or manually through structural alignments.

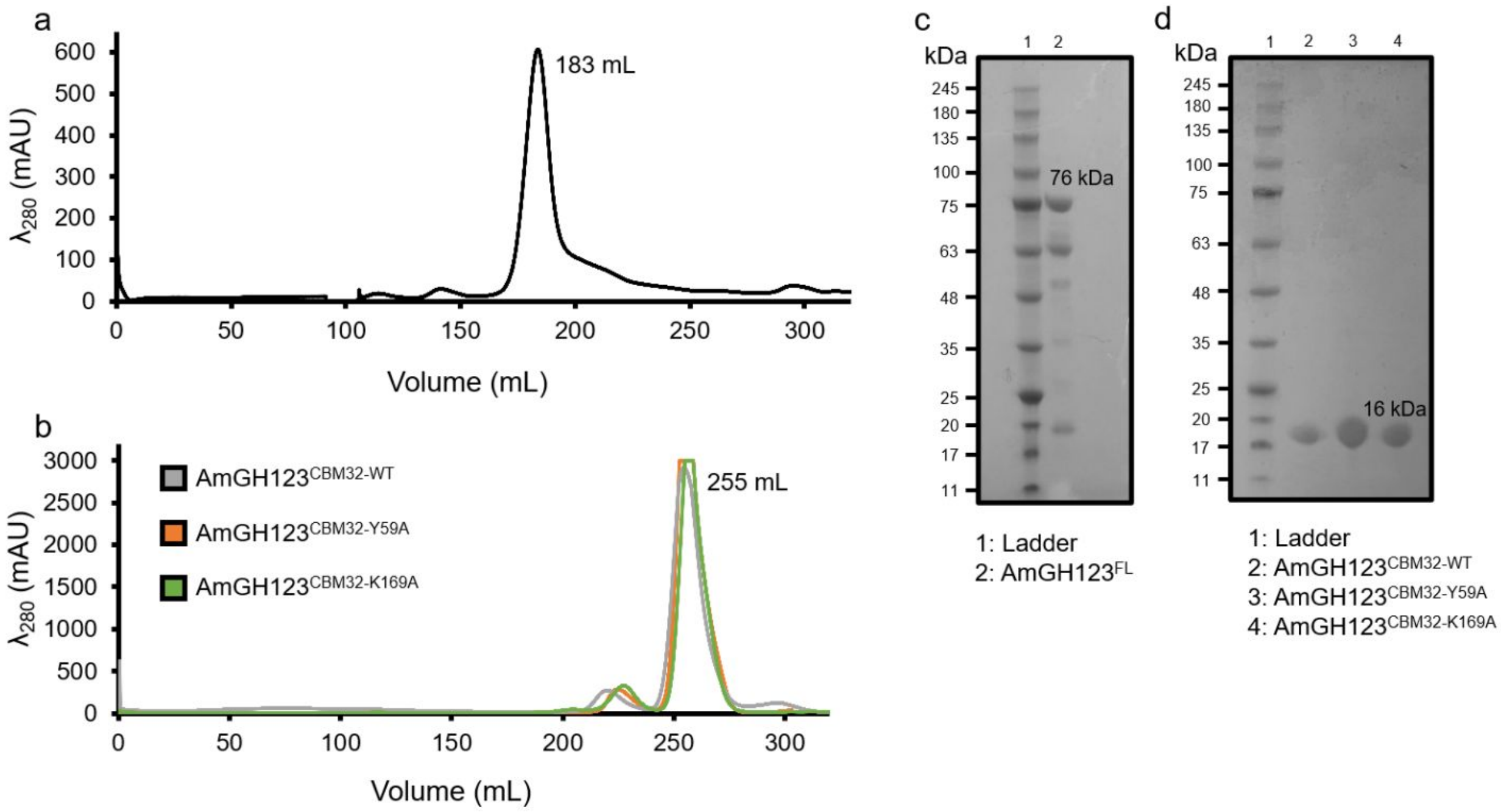

Figure S2: Purified AmGH123^{FL}, AmGH123^{CBM32} wild type, AmGH123$^{CBM32\text{-}Y59A}$, and AmGH123$^{CBM32\text{-}K169A}$. Size exclusion chromatography profiles for (**a**) AmGH123^{FL}, and (**b**) AmGH123$^{CBM32\text{-}WT}$ (grey), AmGH123$^{CBM32\text{-}Y59A}$ (orange) and AmGH123$^{CBM32\text{-}K169A}$ (green). SDS-Page gels for all purified constructs (**c**) AmGH123^{FL}, and (**d**) AmGH123$^{CBM32\text{-}WT}$, AmGH123$^{CBM32\text{-}Y59A}$ and AmGH123$^{CBM32\text{-}K169A}$. AmGH123^{FL} appears to undergo partial fragmentation during purification, resulting in separation of the catalytic and CBM modules.

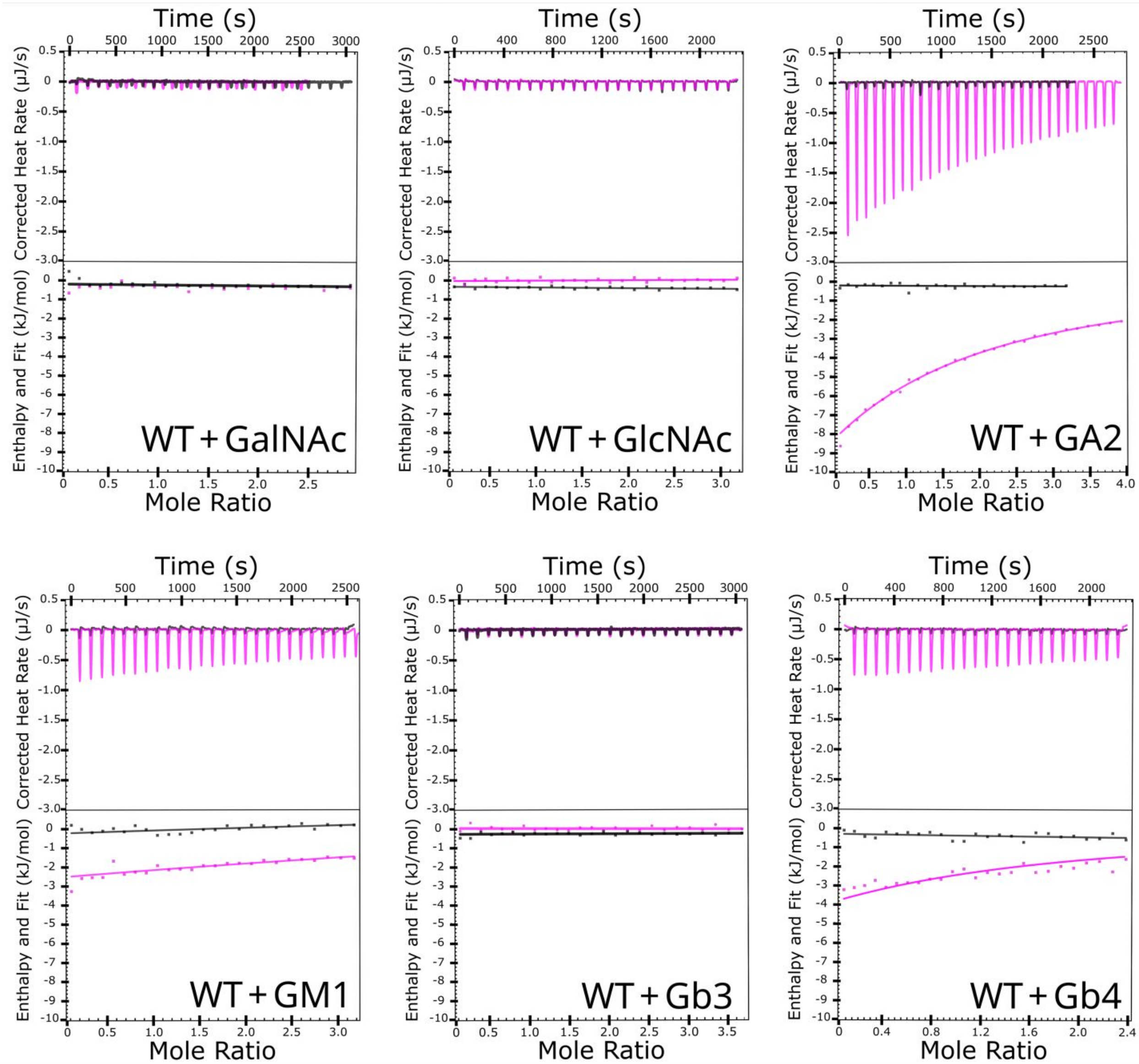

Figure S3: Representative ITC data for AmGH123$^{CBM32\text{-}WT}$ with GalNAc, GlcNAc, globotriose (Gb3), gangliotriose (GA2), globo-N-tetraose (Gb4), and gangliotetraose (GM1)**.**

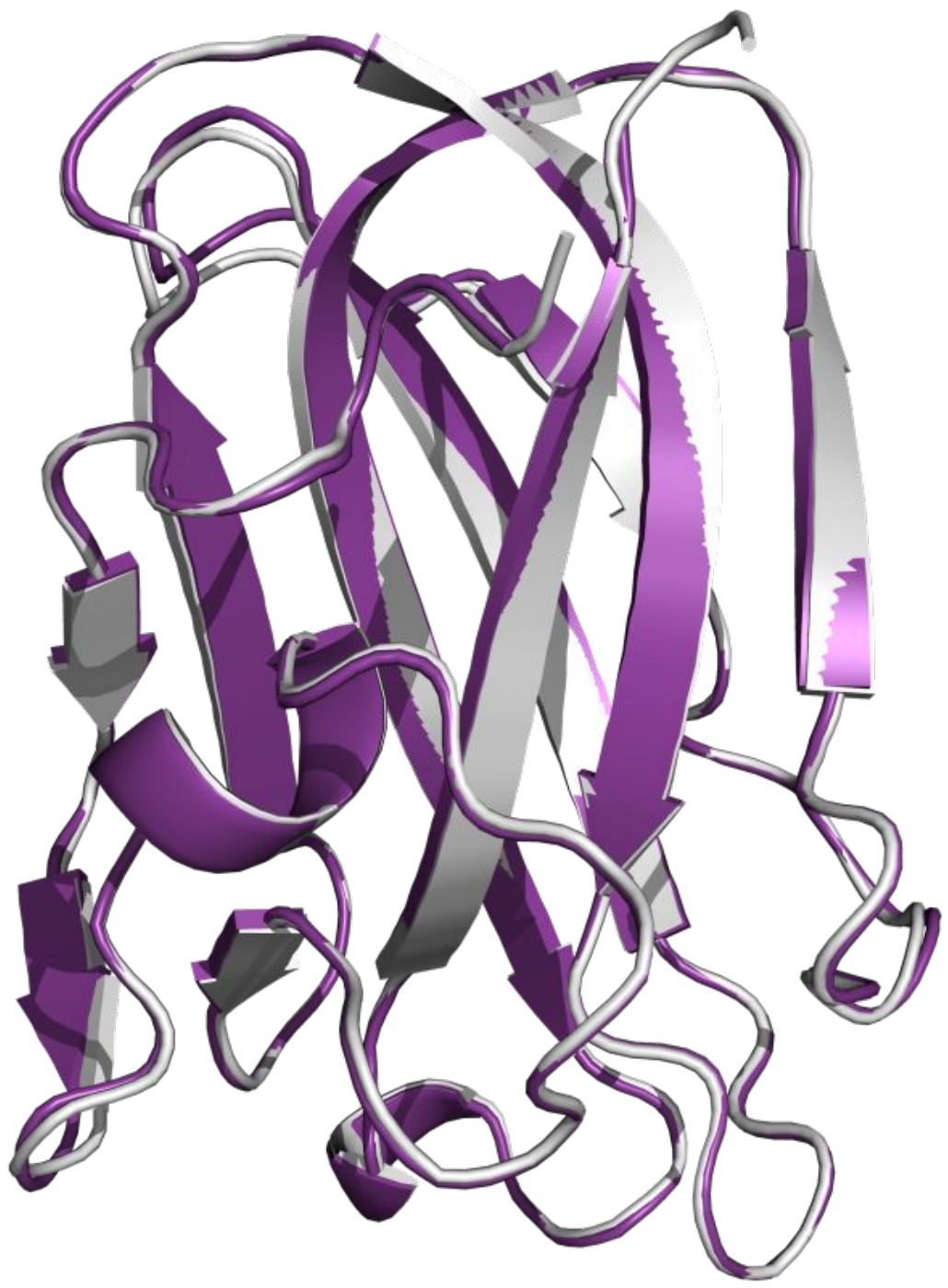

Figure S4: Comparison of AmGH123^{CBM32} chain A (grey) and chain B (purple) structures.

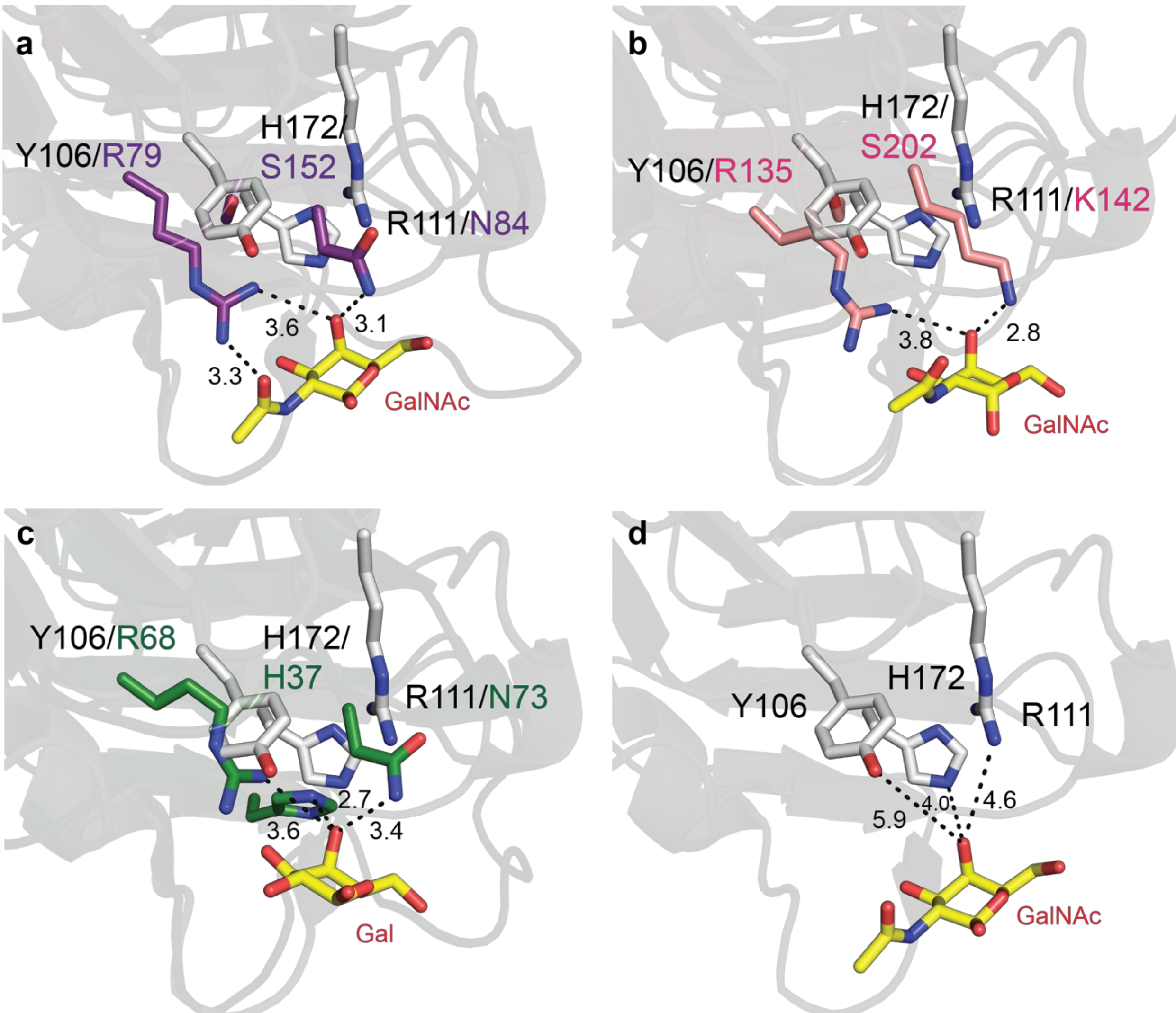

Figure S5: Structural comparison of AmGH123^{CBM32} (grey) with **a)** CpGH31$^{CBM32-3}$ (purple; PDB ID 4P5Y), **b)** CpZmpB^{CBM32} (pink; PDB ID 7JNF), and **c)** CpGH33^{CBM32} (green; PDB ID 2V72). Gal and GalNAc residues are shown as yellow sticks. Residues are labeled with the AmGH123^{CBM32} residue in black followed by the corresponding residue from the comparison CBM32 in its respective color. **d)** AmGH123^{CBM32} modeled with the GalNAc ligand from CpGH31$^{CBM32-3}$. Potential H-bonding distances are shown in Å. Oxygen and nitrogen atoms are colored red and blue, respectively.

Comparison with structurally characterized Gal- and GalNAc-binding CBM32 members reveals a recurring role for interactions with the C4 hydroxyl of galacto-configured sugars, which may contribute to discrimination against gluco-configured ligands (which differs only in

configuration at the C4 position). However, the residues involved in C4 recognition vary among CBM32 family members. In CpGH31$^{\text{CBM32-3}}$, R79 and N84 are positioned to interact with the C4 hydroxyl (**Figure S5a**), whereas the corresponding positions in CpZmpB$^{\text{CBM32}}$ are occupied by R135 and K142 (**Figure S5b**). In CpGH33$^{\text{CBM32}}$, R68, N73, and H37 contribute to a similar interaction network around the C4 hydroxyl (**Figure S5c**). In AmGH123$^{\text{CBM32}}$, the equivalent positions are occupied by Y106, R111, and H172, respectively (**Figure S5d**). Thus, although H172 occupies a position similar to H37 in CpGH33$^{\text{CBM32}}$, the Arg present in the other CBM32 members is replaced by Y106, while R111 occupies the position corresponding to N84, K142, or N73. Moreover, in the modeled AmGH123$^{\text{CBM32}}$ complex, these residues are too distant to form obvious direct hydrogen bonds with the C4 hydroxyl. Given the variability in ligand orientation and binding-site architecture across CBM32 members, the structural basis for galacto-specificity in AmGH123$^{\text{CBM32}}$ cannot be confidently assigned from the current model.

Recognition of the GalNAc N-acetyl group is similarly variable among CBM32 members. In CpGH31$^{\text{CBM32-3}}$ and CpZmpB$^{\text{CBM32}}$, the acetamido group is positioned near R79 and R135, respectively, providing potential interactions with this substituent. CpGH33$^{\text{CBM32}}$ binds both Gal and GalNAc but has only been structurally characterized in complex with Gal, preventing direct assessment of N-acetyl recognition; however, the corresponding Arg is conserved among these three CBM32s and could potentially contribute to GalNAc recognition. In contrast, AmGH123$^{\text{CBM32}}$ contains Y106 at this position, and in our modeled GalNAc complex the N-acetyl group is solvent exposed without an obvious direct interaction with a protein side chain (**Figure 3c**). Thus, although AmGH123$^{\text{CBM32}}$ shows a clear requirement for terminal GalNAc, with no detectable binding to corresponding terminal Gal-containing oligosaccharides, the molecular basis for recognition of the N-acetyl group cannot be readily explained by the current apo structure and ligand model.

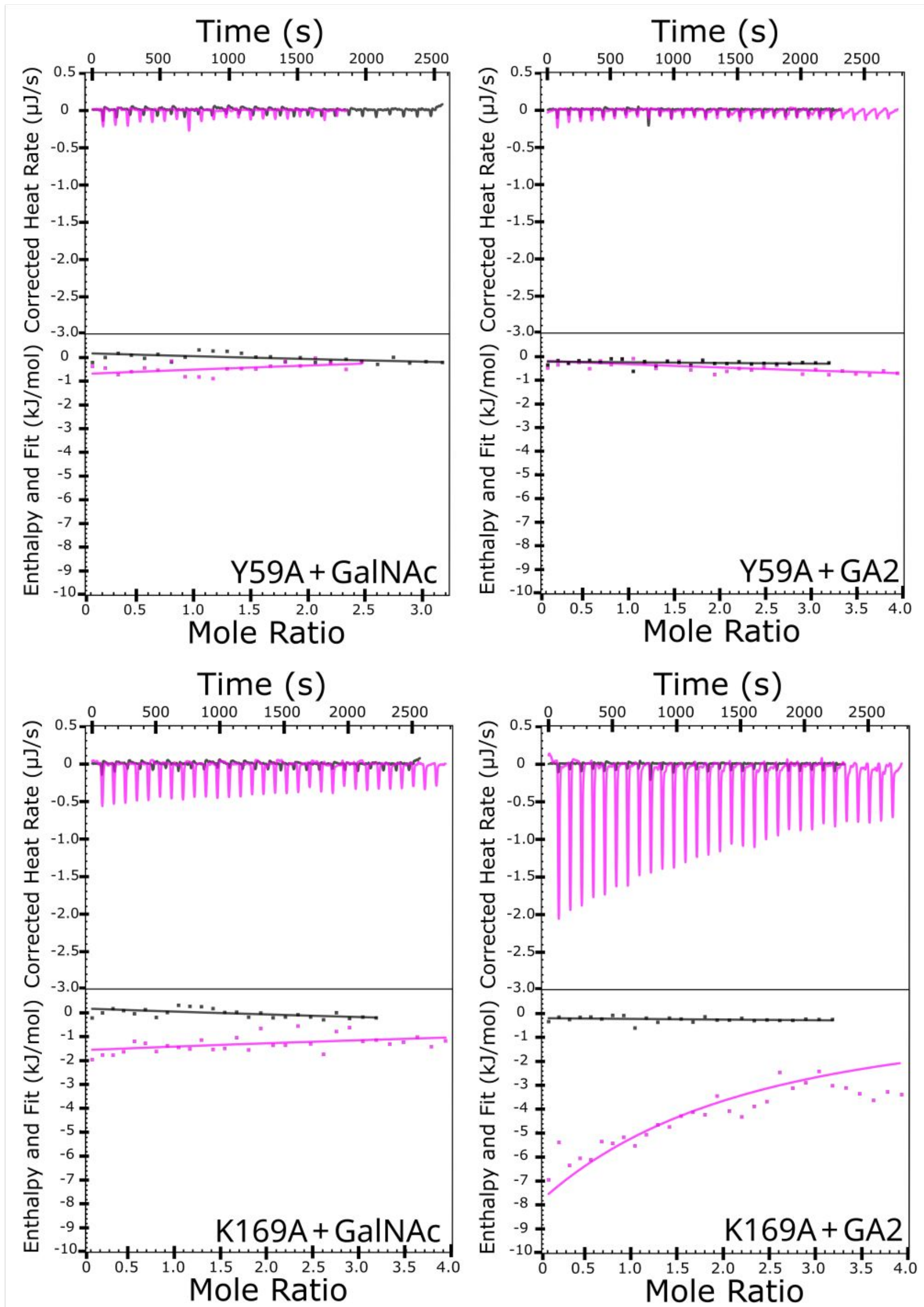

Figure S6: Representative ITC data for AmGH123$^{CBM32-Y59A}$ and AmGH123$^{CBM32-K169A}$ variants with GalNAc and gangliotriose (GA2).

References:

(1) Lombard, V.; Golaconda Ramulu, H.; Drula, E.; Coutinho, P. M.; Henrissat, B. The Carbohydrate-Active Enzymes Database (CAZy) in 2013. *Nucleic Acids Res.* **2014**, *42* (Database issue), D490-495. https://doi.org/10.1093/nar/gkt1178

(2) The UniProt Consortium. UniProt: The Universal Protein Knowledgebase in 2023. *Nucleic Acids Res.* **2023**, *51* (D1), D523–D531. https://doi.org/10.1093/nar/gkac1052

(3) Paysan-Lafosse, T.; Blum, M.; Chuguransky, S.; Grego, T.; Pinto, B. L.; Salazar, G. A.; Bileschi, M. L.; Bork, P.; Bridge, A.; Colwell, L.; Gough, J.; Haft, D. H.; Letunić, I.; Marchler-Bauer, A.; Mi, H.; Natale, D. A.; Orengo, C. A.; Pandurangan, A. P.; Rivoire, C.; Sigrist, C. J. A.; Sillitoe, I.; Thanki, N.; Thomas, P. D.; Tosatto, S. C. E.; Wu, C. H.; Bateman, A. InterPro in 2022. *Nucleic Acids Res.* **2023**, *51* (D1), D418–D427. https://doi.org/10.1093/nar/gkac993

(4) *An efficient one-step site-directed deletion, insertion, single and multiple-site plasmid mutagenesis protocol | BMC Biotechnology | Full Text*. https://bmcbiotechnol.biomedcentral.com/articles/10.1186/1472-6750-8-91 (accessed 2025-09-03)

(5) Gasteiger, E.; Hoogland, C.; Gattiker, A.; Duvaud, S.; Wilkins, M. R.; Appel, R. D.; Bairoch, A. Protein Identification and Analysis Tools on the ExPASy Server. In *The Proteomics Protocols Handbook*; Walker, J. M., Ed.; Humana Press: Totowa, NJ, 2005; pp 571–607. https://doi.org/10.1385/1-59259-890-0:571

(6) Bowers, G. N., Jr; McComb, R. B.; Christensen, R. G.; Schaffer, R. High-Purity 4-Nitrophenol: Purification, Characterization, and Specifications for Use as a Spectrophotometric Reference Material. *Clin. Chem.* **1980**, *26* (6), 724–729. https://doi.org/10.1093/clinchem/26.6.724

(7) *Diphenylamine–Aniline–Phosphoric Acid Reagent, a Versatile Spray Reagent for Revealing Glycoconjugates on Thin-Layer Chromatography Plates*. https://www.researchgate.net/publication/12216429_Diphenylamine-Aniline-Phosphoric_Acid_Reagent_a_Versatile_Spray_Reagent_for_Revealing_Glycoconjugates_on_Thin-Layer_Chromatography_Plates (accessed 2025-07-31)

(8) Battye, T. G. G.; Kontogiannis, L.; Johnson, O.; Powell, H. R.; Leslie, A. G. W. iMOSFLM: A New Graphical Interface for Diffraction-Image Processing with MOSFLM. *Acta Crystallogr. D Biol. Crystallogr.* **2011**, *67* (Pt 4), 271–281. https://doi.org/10.1107/S0907444910048675

(9) Evans, P. R.; Murshudov, G. N. How Good Are My Data and What Is the Resolution? *Acta Crystallogr. D Biol. Crystallogr.* **2013**, *69* (7), 1204–1214. https://doi.org/10.1107/S0907444913000061

(10) McCoy, A. J.; Grosse-Kunstleve, R. W.; Adams, P. D.; Winn, M. D.; Storoni, L. C.; Read, R. J. Phaser Crystallographic Software. *J. Appl. Crystallogr.* **2007**, *40* (Pt 4), 658–674. https://doi.org/10.1107/S0021889807021206

(11) *AlphaFold Protein Structure Database: massively expanding the structural coverage of protein-sequence space with high-accuracy models | Nucleic Acids Research | Oxford Academic*. https://academic.oup.com/nar/article/50/D1/D439/6430488?login=false (accessed 2023-09-26)

(12) Terwilliger, T. C.; Grosse-Kunstleve, R. W.; Afonine, P. V.; Moriarty, N. W.; Zwart, P. H.; Hung, L. W.; Read, R. J.; Adams, P. D. Iterative Model Building, Structure Refinement and Density Modification with the PHENIX AutoBuild Wizard. *Acta Crystallogr. D Biol. Crystallogr.* **2008**, *64* (Pt 1), 61–69. https://doi.org/10.1107/S090744490705024X

(13) Emsley, P.; Lohkamp, B.; Scott, W. G.; Cowtan, K. Features and Development of Coot. *Acta Crystallogr. D Biol. Crystallogr.* **2010**, *66* (4), 486–501. https://doi.org/10.1107/S0907444910007493

(14) Lovell, S. C.; Davis, I. W.; Arendall, W. B.; de Bakker, P. I. W.; Word, J. M.; Prisant, M. G.; Richardson, J. S.; Richardson, D. C. Structure Validation by Calpha Geometry: Phi,Psi and Cbeta Deviation. *Proteins* **2003**, *50* (3), 437–450. https://doi.org/10.1002/prot.10286
(15) He, X.; Zhao, L.; Tian, Y.; Li, R.; Chu, Q.; Gu, Z.; Zheng, M.; Wang, Y.; Li, S.; Jiang, H.; Jiang, Y.; Wen, L.; Wang, D.; Cheng, X. Highly Accurate Carbohydrate-Binding Site Prediction with DeepGlycanSite. *Nat. Commun.* **2024**, *15* (1), 5163. https://doi.org/10.1038/s41467-024-49516-2
(16) Merkel, D. Docker: Lightweight Linux Containers for Consistent Development and Deployment. *Linux J* **2014**, *2014* (239), 2:2.
(17) *python - Official Image | Docker Hub*. https://hub.docker.com/_/python (accessed 2026-02-13)
(18) Bugnon, M.; Röhrig, U. F.; Goullieux, M.; Perez, M. A. S.; Daina, A.; Michielin, O.; Zoete, V. SwissDock 2024: Major Enhancements for Small-Molecule Docking with Attracting Cavities and AutoDock Vina. *Nucleic Acids Res.* **2024**, *52* (W1), W324–W332. https://doi.org/10.1093/nar/gkae300
(19) Grosdidier, A.; Zoete, V.; Michielin, O. SwissDock, a Protein-Small Molecule Docking Web Service Based on EADock DSS. *Nucleic Acids Res.* **2011**, *39* (suppl_2), W270–W277. https://doi.org/10.1093/nar/gkr366
(20) Grime, G. W.; Zeldin, O. B.; Snell, M. E.; Lowe, E. D.; Hunt, J. F.; Montelione, G. T.; Tong, L.; Snell, E. H.; Garman, E. F. High-Throughput PIXE as an Essential Quantitative Assay for Accurate Metalloprotein Structural Analysis: Development and Application. *J. Am. Chem. Soc.* **2020**, *142* (1), 185–197. https://doi.org/10.1021/jacs.9b09186
(21) Cartmell, A.; Muñoz-Muñoz, J.; Briggs, J. A.; Ndeh, D. A.; Lowe, E. C.; Baslé, A.; Terrapon, N.; Stott, K.; Heunis, T.; Gray, J.; Yu, L.; Dupree, P.; Fernandes, P. Z.; Shah, S.; Williams, S. J.; Labourel, A.; Trost, M.; Henrissat, B.; Gilbert, H. J. A Surface Endogalactanase in Bacteroides Thetaiotaomicron Confers Keystone Status for Arabinogalactan Degradation. *Nat. Microbiol.* **2018**, *3* (11), 1314–1326. https://doi.org/10.1038/s41564-018-0258-8
(22) Katoh, T.; Yamada, C.; Wallace, M. D.; Yoshida, A.; Gotoh, A.; Arai, M.; Maeshibu, T.; Kashima, T.; Hagenbeek, A.; Ojima, M. N.; Takada, H.; Sakanaka, M.; Shimizu, H.; Nishiyama, K.; Ashida, H.; Hirose, J.; Suarez-Diez, M.; Nishiyama, M.; Kimura, I.; Stubbs, K. A.; Fushinobu, S.; Katayama, T. A Bacterial Sulfoglycosidase Highlights Mucin O-Glycan Breakdown in the Gut Ecosystem. *Nat. Chem. Biol.* **2023**, *19* (6), 778–789. https://doi.org/10.1038/s41589-023-01272-y
(23) Zhang, X.; Sunagawa, N.; Kashima, T.; Igarashi, K.; Miyanaga, A.; Fushinobu, S. Structural Insights into Lacto- *N* -biose I Recognition by a Family 32 Carbohydrate-binding Module from *Bifidobacterium Bifidum*. *FEBS Lett.* **2026**, *600* (4), 504–514. https://doi.org/10.1002/1873-3468.70217
(24) Ernst, P.; Plückthun, A.; Mittl, P. R. E. Structural Analysis of Biological Targets by Host:Guest Crystal Lattice Engineering. *Sci. Rep.* **2019**, *9* (1), 15199. https://doi.org/10.1038/s41598-019-51017-y
(25) Grondin, J. M.; Chitayat, S.; Ficko-Blean, E.; Houliston, S.; Arrowsmith, C. H.; Boraston, A. B.; Smith, S. P. An Unusual Mode of Galactose Recognition by a Family 32 Carbohydrate-Binding Module. *J. Mol. Biol.* **2014**, *426* (4), 869–880. https://doi.org/10.1016/j.jmb.2013.11.029
(26) Ficko-Blean, E.; Boraston, A. B. N-Acetylglucosamine Recognition by a Family 32 Carbohydrate-Binding Module from Clostridium Perfringens NagH. *J. Mol. Biol.* **2009**, *390* (2), 208–220. https://doi.org/10.1016/j.jmb.2009.04.066
(27) Ficko-Blean, E.; Boraston, A. B. The Interaction of a Carbohydrate-Binding Module from a Clostridium Perfringens N-Acetyl-β-Hexosaminidase with Its Carbohydrate Receptor. *J. Biol. Chem.* **2006**, *281* (49), 37748–37757. https://doi.org/10.1074/jbc.M606126200

(28) Grondin, J. M.; Duan, D.; Kirlin, A. C.; Abe, K. T.; Chitayat, S.; Spencer, H. L.; Spencer, C.; Campigotto, A.; Houliston, S.; Arrowsmith, C. H.; Allingham, J. S.; Boraston, A. B.; Smith, S. P. Diverse Modes of Galacto-Specific Carbohydrate Recognition by a Family 31 Glycoside Hydrolase from Clostridium Perfringens. *PLOS ONE* **2017**, *12* (2), e0171606. https://doi.org/10.1371/journal.pone.0171606
(29) Ficko-Blean, E.; Stubbs, K. A.; Nemirovsky, O.; Vocadlo, D. J.; Boraston, A. B. Structural and Mechanistic Insight into the Basis of Mucopolysaccharidosis IIIB. *Proc. Natl. Acad. Sci.* **2008**, *105* (18), 6560–6565. https://doi.org/10.1073/pnas.0711491105
(30) Ficko-Blean, E.; Stuart, C. P.; Suits, M. D.; Cid, M.; Tessier, M.; Woods, R. J.; Boraston, A. B. Carbohydrate Recognition by an Architecturally Complex α-N-Acetylglucosaminidase from Clostridium Perfringens. *PLoS ONE* **2012**, *7* (3), e33524. https://doi.org/10.1371/journal.pone.0033524
(31) Pluvinage, B.; Ficko-Blean, E.; Noach, I.; Stuart, C.; Thompson, N.; McClure, H.; Buenbrazo, N.; Wakarchuk, W.; Boraston, A. B. Architecturally Complex *O* -Glycopeptidases Are Customized for Mucin Recognition and Hydrolysis. *Proc. Natl. Acad. Sci.* **2021**, *118* (10), e2019220118. https://doi.org/10.1073/pnas.2019220118
(32) Boraston, A. B.; Ficko-Blean, E.; Healey, M. Carbohydrate Recognition by a Large Sialidase Toxin from *Clostridium Perfringens*. *Biochemistry* **2007**, *46* (40), 11352–11360. https://doi.org/10.1021/bi701317g
(33) Wu, H.; Rebello, O.; Crost, E. H.; Owen, C. D.; Walpole, S.; Bennati-Granier, C.; Ndeh, D.; Monaco, S.; Hicks, T.; Colvile, A.; Urbanowicz, P. A.; Walsh, M. A.; Angulo, J.; Spencer, D. I. R.; Juge, N. Fucosidases from the Human Gut Symbiont Ruminococcus Gnavus. *Cell. Mol. Life Sci.* **2021**, *78* (2), 675–693. https://doi.org/10.1007/s00018-020-03514-x
(34) Gaskell, A.; Crennell, S.; Taylor, G. The Three Domains of a Bacterial Sialidase: A β-Propeller, an Immunoglobulin Module and a Galactose-Binding Jelly-Roll. *Structure* **1995**, *3* (11), 1197–1205. https://doi.org/10.1016/S0969-2126(01)00255-6
(35) Kołaczkowski, B. M.; Moroz, O. V.; Blagova, E.; Davies, G. J.; Møller, M. S.; Meyer, A. S.; Westh, P.; Jensen, K.; Wilson, K. S.; Krogh, K. B. R. M. Structural and Functional Characterization of a Multi-Domain GH92 α-1,2-Mannosidase from *Neobacillus Novalis*. *Acta Crystallogr. Sect. Struct. Biol.* **2023**, *79* (5), 387–400. https://doi.org/10.1107/S2059798323001663
(36) Shinya, S.; Nishimura, S.; Kitaoku, Y.; Numata, T.; Kimoto, H.; Kusaoke, H.; Ohnuma, T.; Fukamizo, T. Mechanism of Chitosan Recognition by CBM32 Carbohydrate-Binding Modules from a *Paenibacillus* Sp. IK-5 Chitosanase/Glucanase. *Biochem. J.* **2016**, *473* (8), 1085–1095. https://doi.org/10.1042/BCJ20160045
(37) Teh, A.-H.; Sim, P.-F.; Hisano, T. Structural Basis for Binding Uronic Acids by Family 32 Carbohydrate-Binding Modules. *Biochem. Biophys. Res. Commun.* **2020**, *533* (3), 257–261. https://doi.org/10.1016/j.bbrc.2020.09.064
(38) Abbott, D. W.; Boraston, A. Structural Analysis of a Putative Family 32 Carbohydrate-Binding Module from the *Streptococcus Pneumoniae* Enzyme EndoD. *Acta Crystallograph. Sect. F Struct. Biol. Cryst. Commun.* **2011**, *67* (4), 429–433. https://doi.org/10.1107/S1744309111001874
(39) Zhang, K.; Li, Z.; Zhu, Q.; Cao, H.; He, X.; Zhang, X.-H.; Liu, W.; Lyu, Q. Determination of Oligosaccharide Product Distributions of PL7 Alginate Lyases by Their Structural Elements. *Commun. Biol.* **2022**, *5* (1), 782. https://doi.org/10.1038/s42003-022-03721-1
(40) Abbott, D. W.; Hrynuik, S.; Boraston, A. B. Identification and Characterization of a Novel Periplasmic Polygalacturonic Acid Binding Protein from Yersinia Enterolitica. *J. Mol. Biol.* **2007**, *367* (4), 1023–1033. https://doi.org/10.1016/j.jmb.2007.01.030
(41) Summers, E. L.; Moon, C. D.; Atua, R.; Arcus, V. L. The Structure of a Glycoside Hydrolase 29 Family Member from a Rumen Bacterium Reveals Unique, Dual

Carbohydrate-Binding Domains. *Acta Crystallogr. Sect. F Struct. Biol. Commun.* **2016**, *72* (10), 750–761. https://doi.org/10.1107/S2053230X16014072
